# The Impact of Long-term Physical Inactivity on Adipose Tissue Immunometabolism

**DOI:** 10.1210/clinem/dgab647

**Published:** 2021-09-04

**Authors:** William V Trim, Jean-Philippe Walhin, Francoise Koumanov, Anne Bouloumié, Mark A Lindsay, Rebecca L Travers, James E Turner, Dylan Thompson

**Affiliations:** 1 Centre for Nutrition, Exercise and Metabolism (CNEM), Department for Health, University of Bath, Bath, UK; 2 INSERM, I2MC, Toulouse, France; 3 Department of Pharmacy and Pharmacology, University of Bath, Bath, UK

**Keywords:** Adipose tissue, physical inactivity, immunometabolism

## Abstract

**Context:**

Adipose tissue and physical inactivity both influence metabolic health and systemic inflammation, but how adipose tissue responds to chronic physical inactivity is unknown.

**Objective:**

This work aimed to characterize the impact of chronic physical inactivity on adipose tissue in healthy, young males.

**Methods:**

We collected subcutaneous adipose tissue from 20 healthy, young men before and after 60 days of complete bed rest with energy intake reduced to maintain energy balance and fat mass. We used RNA sequencing, flow cytometry, ex vivo tissue culture, and targeted protein analyses to examine adipose tissue phenotype.

**Results:**

Our results indicate that the adipose tissue transcriptome, stromal cellular compartment, and insulin signaling protein abundance are largely unaffected by bed rest when fat mass is kept stable. However, there was an increase in the circulating concentration of several adipokines, including plasma leptin, which was associated with inactivity-induced increases in plasma insulin and absent from adipose tissue cultured ex vivo under standardized culture conditions.

**Conclusion:**

Physical inactivity–induced disturbances to adipokine concentrations such as leptin, without changes to fat mass, could have profound metabolic implications outside a clinical facility when energy intake is not tightly controlled.

Adipose tissue is an endocrine organ that contributes to systemic inflammation in obesity (1), and influences the function of other tissues, including muscle and bone (2). Indeed, interleukin (IL)-6, tumor necrosis factor (TNF)-α, and monocyte chemoattractant protein (MCP)-1 released either from adipocytes or cells of the adipose stromal vascular fraction (SVF) are implicated in muscle insulin resistance and lipotoxicity (3, 4), whereas leptin and adiponectin modulate muscle fat oxidation and appetite control, and influence insulin sensitivity (5, 6). Adipose tissue dysfunction drives local and systemic inflammatory and metabolic dysregulation, contributing to the development of insulin resistance and type 2 diabetes (7).

It is not clear how chronic physical inactivity impacts adipose tissue physiology. Bed rest is a useful model for physical inactivity, which is also used by space agencies as a ground-based model for microgravity. Although adipose tissue responses to chronic to bed rest are understudied, changes in musculoskeletal and systemic outcomes are well characterized, including lean mass (8, 9), insulin sensitivity (10, 11), lipid handling capacity (12, 13), and measures of systemic inflammation (14, 15). One investigation reported that subcutaneous adipose tissue glucose uptake, adipose interstitial IL-6 concentrations, and *LPL* mRNA increased after 10 days of bed rest, while lipolysis was reduced by ~20% (16-18). Furthermore, systemic changes in circulating proteins produced by adipose tissue (such as increased leptin and adiponectin) have been reported with both short (7-14 days) and long-term (60 days) bed rest (19-21). Thus, there is some indication that adipose tissue may be affected by physical inactivity, but existing observations come from relatively short-term studies and, to date, none have undertaken a broad characterization of adipose tissue in response to bed rest.

Using RNA sequencing, ex vivo tissue culture, flow cytometry, and targeted protein analysis, we characterized the effect of long-term best rest on adipose tissue inflammatory and metabolic health in healthy, young men over 60 days of bed rest where energy balance was strictly controlled.

## Materials and Methods

### Experimental Design

**Figure 1.**
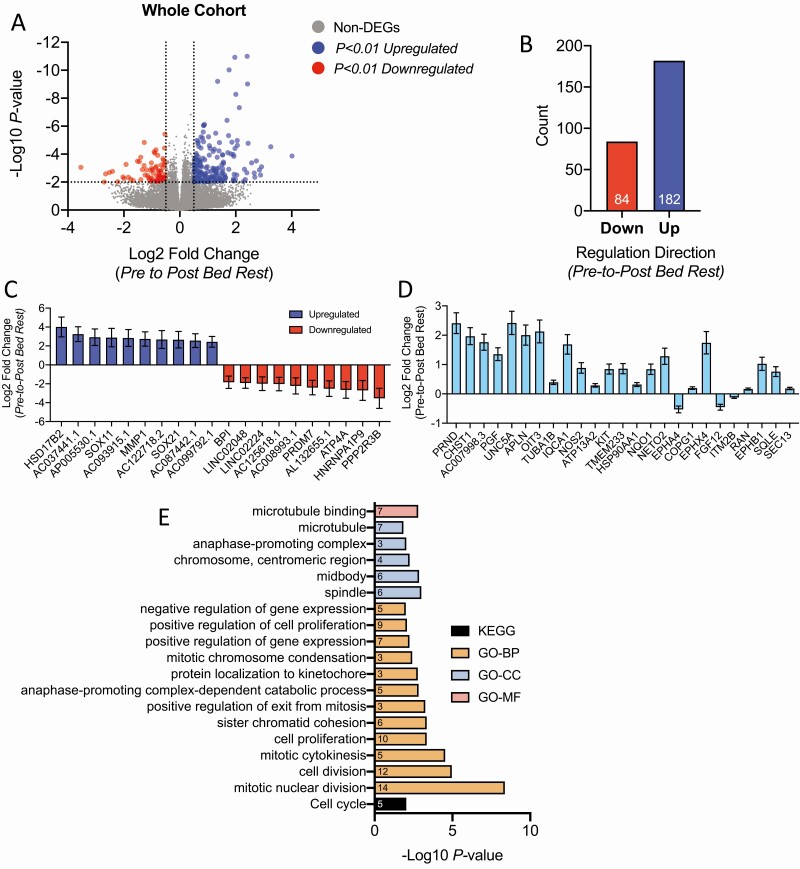
Overview of adipose tissue transcriptional responses to long-term bed rest. (A) Volcano plot of all transcripts by Log2FC against DESeq2-produced p-values. Horizontal dotted lines indicate significance threshold (*P* < .01). Vertical dotted lines represent 0.5 Log2 fold change thresholds. Red dots = significantly downregulated; blue dots = significantly upregulated; grey dots = non-differentially expressed genes. (B) Quantification of differentially expressed genes identified in (A). (C) Top-10 significantly down/upregulated transcripts, by magnitude of Log2FC (presented as mean ± SEM). (D) Top 25 most significantly DEGs ranked by significance, from left to right (presented as mean ± SEM). (E) Significantly enriched (*P* < .05; EASE score) pathways associated with DESeq2 DEGs from (A) using DAVID bioinformatics resources 6.8. *P* values are presented as –Log10 values. Pathways and terms are ranked in ascending order by significance (EASE score). The specific number of differentially expressed genes within a given pathway related to differentially expressed genes put into the analyses are presented within each respective bar. A minimum threshold of 2 genes within a given pathway/term was set for consideration. BP, biological process; CC, cellular compartment; DAVID, database for annotation, visualization, and integrated discovery; DEG, differentially expressed gene; EASE, expression analysis systematic explorer; GO, gene ontology; KEGG, Kyoto encyclopedia of genes and genomes; MF, molecular function.

**Figure 2.**
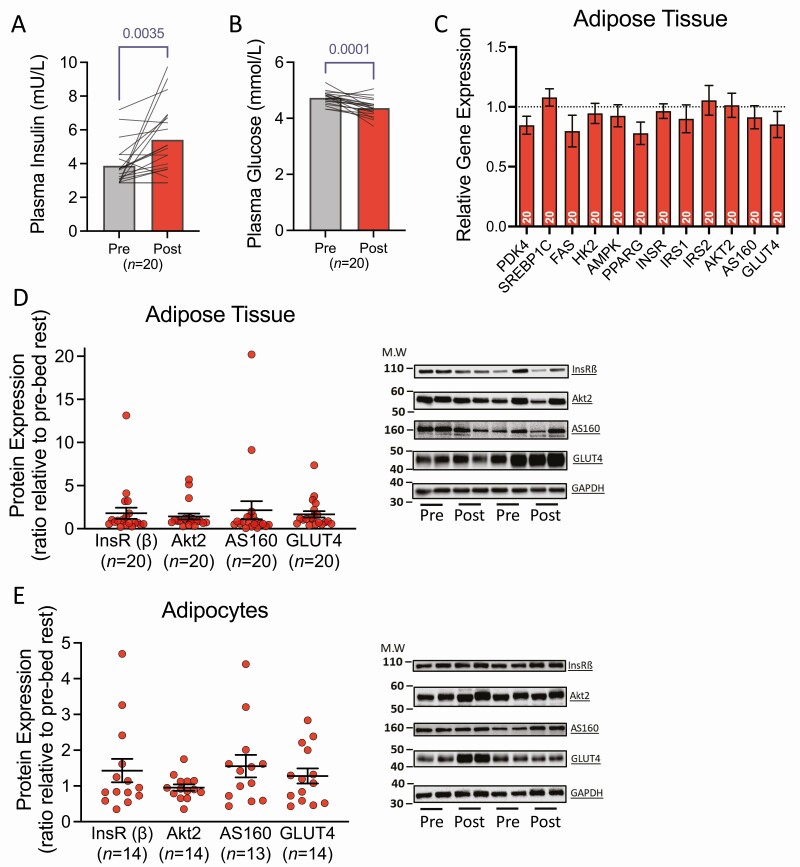
Plasma glucose and insulin, and metabolic gene and protein expression in adipose tissue in response to bed rest. (A) Fasting plasma insulin concentrations before and after bed rest. Data represent group means at each time point with individual responses overlaid. (B) Fasting plasma glucose concentrations before and after bed rest. Data represent group means at each time point with individual responses overlaid. (C) Relative gene expression of several genes associated with adipose tissue metabolism at post bed rest relative to pre bed rest. Ratios represent the fold change at post bed rest compared with pre bed rest expression levels. Data were normalized to peptidylprolyl isomerase A, pre bed rest, and internal calibrator using the ∆∆Ct method. Dashed line represents no change relative to pre bed rest. Data are presented as mean ± SEM, with the sample size indicated at the bottom of each bar. (D, E) Changes in protein expression of key insulin signaling proteins in (D) adipose tissue and (E) paired adipocytes as a ratio of post bed rest compared with pre bed rest levels, with representative blots (right of respective figure). Solid lines represent mean ± SEM, with individual responses overlaid. Dashed lines in Panel C represent no change relative to pre bed rest. Sample sizes are indicated underneath each variable in A, B, D, and E, and within bars in C.

Twenty healthy, young (20-45 years old) men undertook 60 days of complete bed rest (24 hours a day), at a 6° decline, preceded by a 14-day ambulatory control period. The study was conducted in accordance with European Space Agency Standardization of Bed Rest Study Conditions guidelines (22) at the Médecine et de Physiologie Spatiales (MEDES) clinical institute in Toulouse, France. The study was sponsored by the ESA and French National Space Agency (CNES) and was conducted in 2 campaigns (n = 10 per campaign) from January to April 2017 and September to December 2017. The protocol was reviewed and approved by the local ethics committee (CPP Sud-Ouest et Outre-Mer I, France, RCB: 2016-A00401-50) and was registered on ClinicalTrials.gov (NCT03594799). All procedures conformed to the Declaration of Helsinki. Participants were recruited by online advertisements and press announcements. Screening/selection processes and volunteer numbers are presented elsewhere (Figure 1 and Table 1, respectively (23)). A full overview of the experimental procedures is presented elsewhere (Figure 2A (23)).

Within each campaign, half of participants (n = 5 in each campaign) consumed an antioxidant/anti-inflammatory nutritional cocktail during the bed rest period (Cocktail group), and the other half received no supplement (Control group). Group allocation was blinded from all researchers until the completion of each bed rest campaign. The nutritional supplement has been examined previously (24, 25) and described in Table 2 (23). No placebo was administered to the Control group due to the inability to mask the fish oil odor of ω-3 supplementation. Our primary focus was on the impact of bed rest on adipose tissue and there was no evidence of a supplement effect, thus our analysis focuses on the impact of bed rest per se across all study participants (see “Results”).

### Body Composition

Body composition, fat mass index (26), and central fat mass (fat mass between L1 and L4 vertebrae) (27, 28) were determined using dual-energy X-ray absorptiometry (Discovery, Hologic; Bedford, UK) 2 days prior to the start of bed rest and following 58 days of bed rest.

### Physical Activity Baseline Standardization

During the 14-day pre bed rest period in the clinical facility, participants undertook approximately 8000 steps/day measured with a wrist-mounted accelerometer (Polar Loop; Polar; France). Participants also undertook bouts of supervised structured exercise during this standardization period on a treadmill and cycling ergometer (see Figure 2A (23)).

### Dietary Control

Diet was strictly controlled and recorded throughout the study period. Macro- and micronutrient intake for the pre bed rest and bed rest period are presented in Table 3 (23). Energy intake was based on basal metabolic rate (BMR) measured by indirect calorimetry (22). During the pre bed rest period 140% of BMR was consumed, which was reduced to 110% BMR during the bed rest period in order to keep fat mass stable (22). An additional 1000 IU of 25 (OH) vitamin D was supplemented daily by oral administration as specified by Heer et al. (22).

### Blood Sampling

Fasted venous blood samples were collected from an antecubital vein at 07:00 hours 6 days prior to the start of bed rest and following 56 days of bed rest. Plasma samples were immediately centrifuged and stored at −80°C. Peripheral blood mononuclear cells (PBMCs) were isolated by density gradient separation (Ficoll®, Greiner Bio-One; Stonehouse, UK) in Leucosep® tubes (Greiner Bio One Inc.; Kremsmünster, Austria) for analysis on the day of collection.

### Adipose Tissue Sampling

Pre and post bed rest subcutaneous adipose tissue samples were obtained ~5 cm lateral to the umbilicus with a 14G needle using the needle aspiration method (28) under local anesthesia (1% lidocaine hydrochloride containing 0.005 mg/mL adrenaline (epinephrine); xylocaine; Dublin, Ireland). Adipose tissue was taken following blood draws 6 days prior to the start of bed rest and following 56 days of bed rest. Adipose samples were extensively cleaned with 0.9% NaCl solution (B. Braun; Sheffield, UK) and were either snap frozen in liquid nitrogen and stored at −80°C, or placed into unsupplemented endothelial cell basal medium (PromoCell; Heidelberg, Germany) at room temperature for tissue culture or enzymatic digestion. Adipose tissue partitioning is detailed in Figure 2B (23).

### Ex Vivo Adipose Tissue Measures

Adipose tissue explants were cultured ex vivo at a final concentration of 50 mg of tissue per milliliter for 3 hours as previously described (29, 30).

### Adipose Tissue Digestion

Between 200 and 500 mg of adipose tissue was digested using collagenase as previously described (28, 30). Isolated adipocytes were recovered by flotation and the SVF cells were recovered following centrifugation at 300*g* for 5 minutes.

### Adipose Tissue RNA Isolation

Total RNA (including microRNAs) was extracted using miRNeasy Mini Kit (Qiagen; Crawley, UK) according to the manufacturer’s instructions. Following RNA isolation, samples were DNase-treated in DNase I supplemented with 10% DNase buffer (Qiagen; Crawley, UK) for 10 minutes at room temperature, and purified as previously described (30).

### Quantitative Polymerase Chain Reaction

Quantitative polymerase chain reaction (qPCR) analysis was performed on DNase-treated RNA from adipose tissue on a StepOne™ analyzer (Applied Biosystems; Warrington, UK) using predesigned TaqMan Assays from Applied Biosystems (Invitrogen; CA, USA) for the measurement of *PDK4* (hs00176875_m1), *SREBP1c* (hs01088691_m1), *AKT2* (hs01086099_m1), *INSR* (hs00961557_m1), *GLUT4* (hs00168966_m1), *IRS2* (hs00275843_s1), *AMPK1*/*2* (hs01562315_m1 and hs00178903_m1), *AS160* (hs00952765_m1), *FAS* (hs00188012_m1), *HK2* (hs00606086_m1), *IRS1* (hs00178563_m1), and *PPARG* (hs01115513_m1). Data were normalized to an internal calibrator (peptidylprolyl isomerase A (31); hc04194521_s1), using the ΔΔ comparative threshold (Ct) method (32).

### Transcriptomic and Bioinformatics Analyses

RNA sequencing was performed on polyA-enriched total RNA, on a HiSeq4000 (Illumina, Inc.; CA, USA) by the Oxford Genomics Centre (Wellcome Trust; Oxford, UK). In brief, total RNA was quantified using RiboGreen (Invitrogen; CA, USA) on the FLUOstar OPTIMA plate reader (BMG Labtech GmbH; Aylesbury, UK) and the size profile and integrity analyzed on the 2200 or 4200 TapeStation (Agilent, RNA ScreenTape; Agilent Technologies, CA, USA). Ribosomal integrity number (RIN) estimates for all samples were between 4 and 8.4. Input material was normalized to 200 ng prior to library preparation. Polyadenylated transcript enrichment and strand-specific library preparation were completed using NEBNext Ultra II mRNA kit (New England Biolabs Inc.; MA, USA) following the manufacturer’s instructions. Libraries were amplified (11 cycles) on a Tetrad (Bio-Rad Laboratories, CA, USA) using in-house unique dual indexing primers (33). Individual libraries were normalized using Qubit, and the size profile was analyzed on the 2200 or 4200 TapeStation. Individual libraries were normalized and pooled together accordingly. The pooled library was diluted to ~10 nmol/L, denatured, and further diluted prior to loading on the sequencer. Paired end sequencing was performed using a HiSeq4000 75 bp platform (Illumina, HiSeq 3000/4000 PE Cluster Kit and 150 cycle SBS Kit),

FastQ sequencing files were processed as previously described (30), using the Galaxy web platform (usegalaxy.org), using the *GRCh38/hg38* reference genome. Transcriptome-wide false discovery rate (FDR) adjustments were applied, with an adjusted significance threshold of *q* < 0.05. Functional annotation was performed in the database for annotation, visualization, and integrated discovery (DAVID) 6.8 (2019 release; (34, 35)) and Genesis 1.8.1 (36). Pathway analysis was performed using Kyoto encyclopedia of genes and genomes (KEGG) and gene ontology (GO) terms, using a modified Fisher exact test (EASE (expression analysis systematic explorer); (37)) with a significance threshold of *P* ≤ .01.

### Immunoblotting

Organic phases were extracted from QIAzol-treated tissue samples and processed for immunoblot analysis as previously described (38). Isolated primary adipocytes were thawed on ice and lysed in radioimmunoprecipitation assay buffer (50 mmol/L Tris [pH 7.4], 150 mmol/L NaCl; 0.5% sodium deoxycholate; 0.1% sodium dodecyl sulfate; 0.1% NP-40), supplemented with HALT™ protease inhibitor cocktail (ThermoFisher™; Leicestershire, UK) and PhosSTOP EASYpack phosphatase inhibitor (Roche AG; Basel, Switzerland).

Proteins were separated by sodium dodecyl sulfate-polyacrylamide gel electrophoresis and transferred to a nitrocellulose membrane for immunoblot analysis using the following antibodies: Akt2 (Cell Signaling Technology; RRID:AB_2225186; https://antibodyregistry.org/AB_2225186; 1 in 500 dilution); Akt substrate of 160 kDa (AS160) (Millipore; RRID:AB_492639; https://antibodyregistry.org/AB_492639; 1 in 500 dilution); glyceraldehyde 3-phosphate dehydrogenase (GAPDH; Proteintech; RRID:AB_2107436; https://antibodyregistry.org/AB_2107436; 1 in 2000 dilution); glucose transporter 4 (GLUT4 (39); 1 in 5000 dilution); and Insulin receptor β-chain (InsRβ; Santa Cruz Biotechnology; RRID:AB_631835; https://antibodyregistry.org/AB_631835; 1 in 500 dilution). Images were acquired with EPI Chemi II darkroom (UVP) and bands were quantified using ImageStudio Lite (LI-COR Biosciences®; NE, USA).

### Flow Cytometry

**Figure 3.**
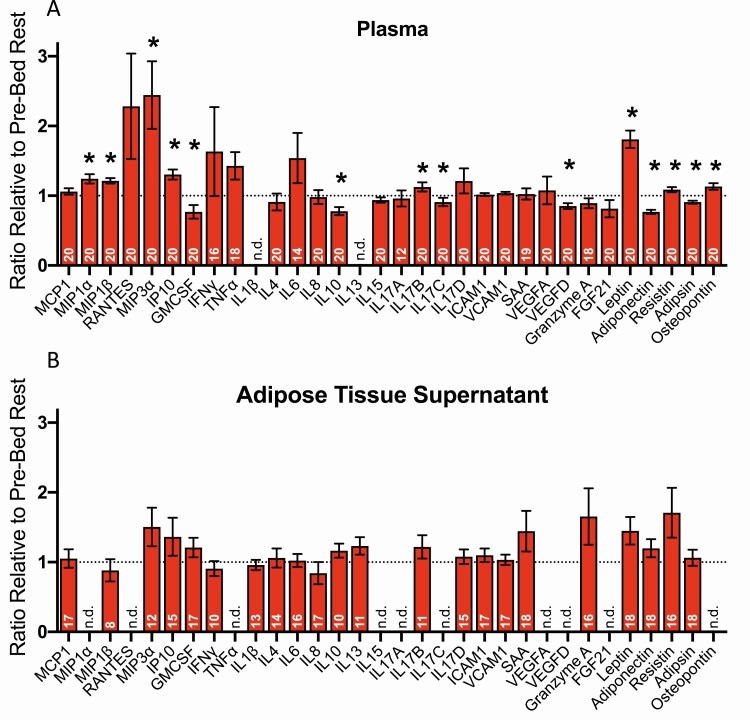
Relative change in plasma and adipose tissue supernatant concentrations of inflammatory and metabolic proteins in response to bed rest. (A) Peripheral blood plasma protein concentration expressed as the average change, calculated from each individual’s change from pre to post bed rest. Data are presented as mean ± SEM. (B) Three-hour ex vivo culture of adipose tissue explant protein secretion expressed as the average change, calculated from each individual’s change from pre to post bed rest. Data are presented as mean ± SEM. Sample sizes are indicated at the base of each respective bar. Where concentrations fell below detectable limits for the assay, n.d. is written. *P < .05. CCL, chemokine (C-C motif) ligand; CXCL, chemokine (C-X-C motif) ligand; FGF, fibroblast growth factor; GM-CSF, granulocyte-macrophage colony stimulating factor; ICAM, intracellular adhesion molecule; IFN, interferon; IL, interleukin; IP, interferon gamma inducible protein; MCP, monocyte chemotactic protein; MIP, monocyte inflammatory protein; RANTES, regulated on activation, normal T-cell expressed and secreted; SAA, serum amyloid-A; TNF, tumor necrosis factor; VCAM, vascular cell adhesion molecule; VEGF, vascular endothelial growth factory.

**Figure 4.**
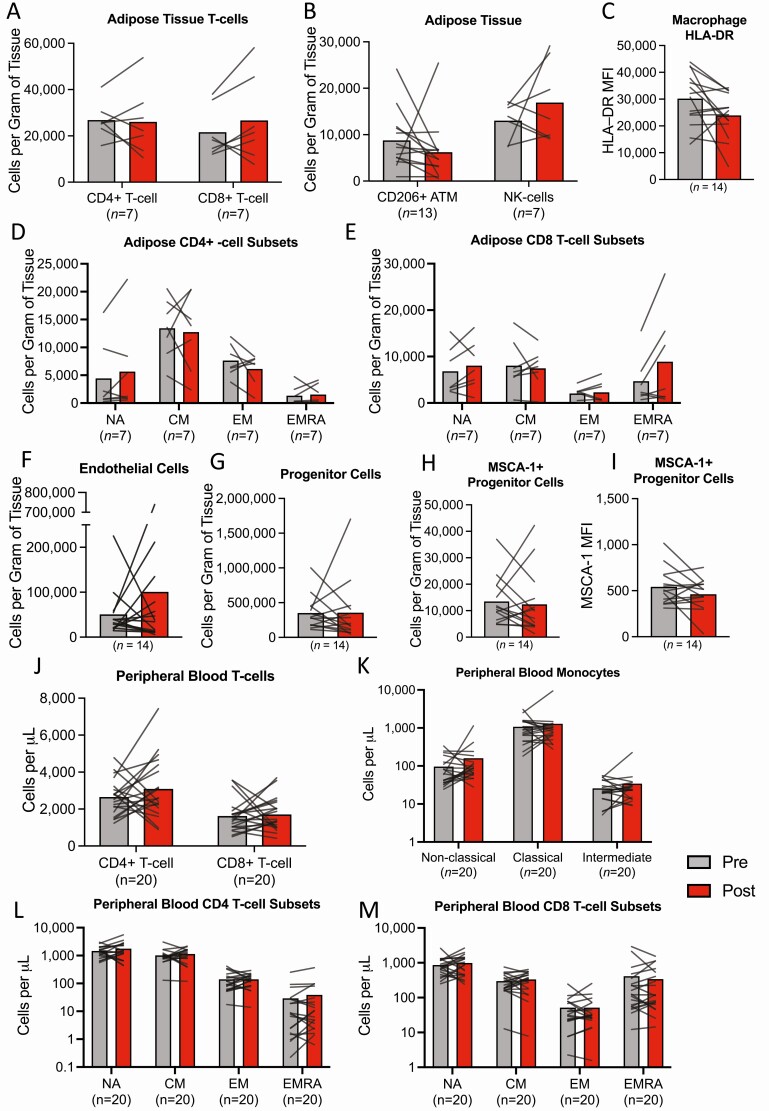
Adipose tissue and peripheral blood leukocyte responses to bed rest. (A) Adipose tissue CD4+ and CD8+ T-cells expressed as number of cells per gram of tissue. (B) Adipose tissue CD206+ macrophages and NK cells expressed as number of cells per gram of tissue. (C) Adipose tissue CD206+ macrophage HLA-DR surface expression (MFI). (D) Adipose tissue CD4+ T-cell subpopulations expressed as number of cells per gram of tissue. (E) Adipose tissue CD8+ T-cell subpopulations expressed as number of cells per gram of tissue. (F) Adipose tissue endothelial cells expressed as number of cells per gram of tissue. (G) Adipose tissue progenitor cells expressed as number of cells per gram of tissue. (H) Adipose tissue MSCA-1+ progenitor cells expressed as number of cells per gram of tissue. (I) Adipose tissue MSCA-1+ progenitor cell MSCA-1+ surface expression (MFI). (J) Peripheral blood CD4+ and CD8+ T-cells expressed as cells per μL. (K) Peripheral blood monocytes subpopulations expressed as cells per μL. (L) Peripheral blood CD4+ T-cell subpopulations expressed as cells per μL. (M) Peripheral blood CD8+ T-cell subpopulations expressed as cells per μL. All data are presented as group means within individual responses overlaid. T-cell subsets presented in (D), (E), (J), and (K) represent CD45RA+CD27+ (NA) cells; CD45RA−CD27+ (CM) cells; CD45RA−CD27− (EM) cells; CD45RA+CD27− (EMRA) cells. Adipose tissue progenitor cells represent CD45−CD31−CD34+ cells. Adipose tissue endothelial cells represent CD45−CD31+CD34+ cells. Sample sizes are indicated underneath each variable. CD, cluster of differentiation; CM, central memory; EM, effector memory; EMRA, effector memory re-expressing CD45RA; NA, naïve; PBMC, peripheral blood mononuclear cell; MFI, median fluorescence intensity; MSCA–1, mesenchymal stem cell antigen-1.

**Figure 5.**
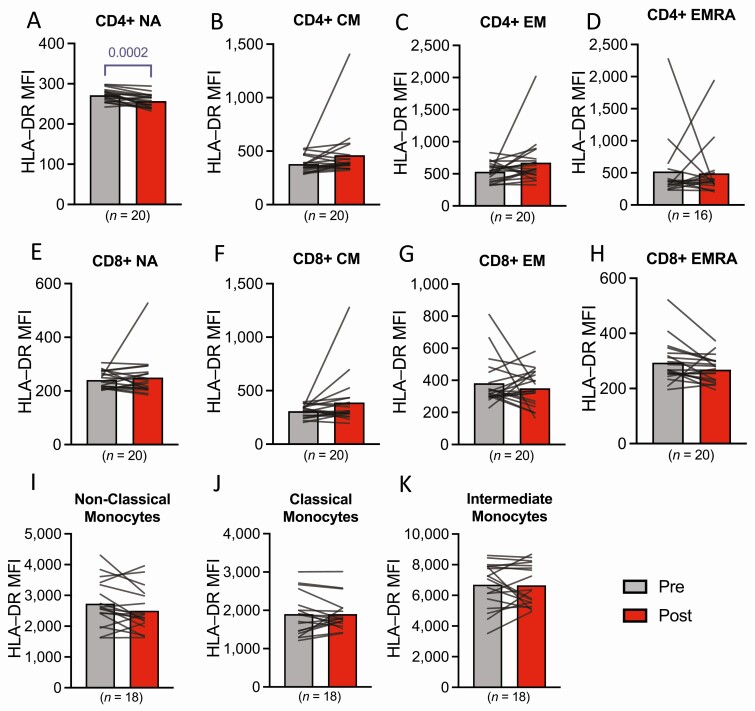
CD4+ T–Cell, CD8+ T–cell, and monocyte subpopulation HLA-DR expression in peripheral blood in response to bed rest. (A-D) CD4+ T-cell subpopulation HLA-DR surface expression for (A) naïve; (B) central memory; (C) effector memory; and (D) terminally differentiated effector memory cells. (E–H) CD8+ T-cell subpopulation HLA-DR surface expression for (E) naïve); (F) central memory; (G) effector memory; and (H) terminally differentiated effector memory cells. (I–K) Monocyte subpopulation HLA-DR surface expression for (I) nonclassical; (J) classical; and (K) intermediate monocytes. T-cell subsets presented in (A-H) represent CD45RA+CD27+ (NA) cells; CD45RA−CD27+ (CM) cells; CD45RA−CD27− (EM) cells; CD45RA+CD27− (EMRA) cells. Data represent HLA-DR cell surface expression on a per-cell basis. Data are presented as group means with individual responses overlaid. Sample sizes are indicated underneath each variable. CD, cluster of differentiation; CM, central memory; EM, effector memory; EMRA, effector memory re-expressing CD45RA; HLA–DR, human leukocyte antigen–DR isotype; MFI, median fluorescence intensity; NA, naïve.

Cells from the SVF were aliquoted into 2 tubes, and 250 000 PBMCs were placed into 2 additional tubes. Samples were split across 2 panels. The T-cell panels contained the following fluorophore-conjugated antibodies: cluster of differentiation (CD) 3–V450 clone UCHT1 (RRID:AB_1645570; https://antibodyregistry.org/AB_1645570), CD4–APC clone RPA–T4 (RRID:AB_398593; https://antibodyregistry.org/AB_398593), CD8–PerCP Sk1 (RRID:AB_400280; https://antibodyregistry.org/AB_400280), CD45RA–FITC clone HI100 (RRID:AB_395879; https://antibodyregistry.org/AB_395879), CD27–PE clone MT271 (RRID:AB_396644; https://antibodyregistry.org/AB_396644), CD45–BV510 clone HI30 (RRID:AB_2738067; https://antibodyregistry.org/AB_2738067), and human leukocyte antigen–DR isotype (HLA–DR)–APC-Cy7 clone L243 (RRID:AB_399974; https://antibodyregistry.org/AB_399974). The monocyte/macrophage panels comprised the following fluorophore-conjugated antibodies: CD14–PE Vio770 clone TUK4 (RRID:AB_2725975; https://antibodyregistry.org/AB_2725975), CD16–FITC clone 3G8 (RRID:AB_396490; https://antibodyregistry.org/AB_396490), CD206–APC clone 19.2 (RRID:AB_398476; https://antibodyregistry.org/AB_398476), HLA-DR–APC Cy7 clone L243 (RRID:AB_399974; https://antibodyregistry.org/AB_399974), and CD45–BV510 clone HI30 (RRID:AB_2738067; https://antibodyregistry.org/AB_2738067). Isotype control antibodies for CD206 (APC–IgG1, κ clone MOPC-21; RRID:AB_398576; https://antibodyregistry.org/AB_398576) and HLA-DR (APC Cy7 IgG2a, κ clone G155-178; RRID:AB_2869659; https://antibodyregistry.org/AB_2869659) were used with PBMCs and informed the gating strategy for both PBMCs and SVF. Endothelial/ progenitor cells were identified in the SVF using the following fluorophore-conjugated antibodies; CD31–V450 clone WM59 (RRID:AB_10896326; https://antibodyregistry.org/AB_10896326), CD34–PerCP clone 8G12 (RRID:AB_400034; https://antibodyregistry.org/AB_400034), and MSCA-1–PE clone W8B2 (RRID:AB_10827708; https://antibodyregistry.org/AB_10827708). Gating strategies are detailed in Figures 3-5 (23). Absolute cell counts were obtained using counting beads (100 µL of Perfect-Count Microspheres; Cytognos, Spain). Flow cytometry was performed on a FACS Canto II (Becton Dickenson; Oxford, UK) and analyzed using FlowJo v.10. Adipose tissue SVF samples that were potentially contaminated by peripheral blood were identified using unbiased outlier assessments detailed in Figure 6 (23).

### Biochemical Analysis of Plasma, Serum, and Ex Vivo Adipose Tissue Supernatants

Fasted plasma insulin was measured by enzyme-linked immunosorbent assay (RRID:AB_2877672; https://antibodyregistry.org/AB_2877672) (Mercodia, Mercodia AB; Sweden). MCP-1, MIP-1α, MIP-1β, RANTES, MIP-3α, IP-10, GM-CSF, IFN-γ, TNF-α, IL-1β, IL-4, IL-6, IL-10, IL-13, IL-15, IL-17A, IL-17B, IL-17C, IL17-D, ICAM-1, VCAM-1, SAA, VEGF-A, VEGF-D, granzyme-A, FGF-21, leptin, adiponectin, resistin, adipsin, and osteopontin were measured in fasted plasma and adipose cell culture supernatant using multiplex assays (R-plex, U-plex and V-plex kits on a QuickPlex SQ120; Mesoscale Diagnostics, LLC; MD, USA). Ex vivo leptin release by adipose tissue was assessed by enzyme-linked immunosorbent assay (Quantikine, Bio-Techne Ltd; France; RRID:AB_2783014; https://antibodyregistry.org/AB_2783014). Blood glucose was measured using fresh whole-blood samples at the clinical facility using an automated analyzer (Architect C8000; Abbott, CA) 4 days prior to the start of bed rest and following 49 days of bed rest.

### Statistical Analysis

Pre to post bed rest comparisons were assessed using paired samples t-tests where data were normally distributed, and Wilcoxon signed rank tests where not normally distributed (Shapiro Wilks: *P* > .05). The effect of bed rest in the Cocktail and Control groups was analyzed using 2-way repeated measures ANOVA. Linear regression analysis was performed using Pearson’s r. Descriptive data are presented as mean ± SD, unless otherwise stated. Statistical analysis was performed using GraphPad Prism v.8.0.0 for Windows (GraphPad Software; CA, USA) and SPSS v.22 (IBM Corp.; NY, USA). Statistical significance was set at *P* ≤ .05.

## Results

### Body Composition Changes in Response to Bed Rest

Participant characteristics are shown in Table 1. In response to bed rest, both total body mass and fat-free mass + bone mineral content decreased by −2.3 ± 1.6 and −5.5 ± 1.6%, respectively. Total fat mass increased modestly ~1 kg (*P* < .0001), while L1-L4 fat mass (as a measure of central adiposity) increased by ~100 g (*P* < .0001) (Table 1).

**Table 1.** Participant characteristics and body composition (DEXA) pre bed rest compared with post bed rest

	Pre bed rest (*N* = 20)	Post bed rest (*N* = 20)	P
Age (years)	34 ± 8	—	—
Height (m)	1.76 ± 0.05	—	—
Body mass (kg)	72.89 ± 7.09	71.17 ± 6.81	<.0001^*a*^
Body mass index (kg/m^2^)	23.5 ± 1.8	22.9 ± 1.8	<.0001^*b*^
Fat-free mass + BMC (kg)	53.69 ± 4.95	50.73 ± 4.45	<.0001^*a*^
Fat mass (kg)	19.20 ± 3.83	20.44 ± 4.08	<.0001^*a*^
Fat mass index (kg/ m^2^)	6.18 ± 1.15	6.58 ± 1.21	<.0001^*a*^
L1-L4 fat mass (kg)	2.24 ± 0.64	2.36 ± 0.68	.0008^*a*^

Body composition data was measured by DEXA. Data represent mean ± SD.

Abbreviations: BMC, bone mineral content; DEXA, dual X-ray absorptiometry; L1-L4, L1-L4 vertebrae.

^*a*^Paired-samples t-tests were performed.

^*b*^Wilcoxon signed-rank tests were performed.

### Adipose Tissue Transcriptomic Responses to Bed Rest

Initial analyses applying a false discovery rate correction built into DESeq2 revealed 42 up- and 9 downregulated transcripts (*q* < 0.05) in response to bed rest (Data File 1 (23)). To further explore these data, all transcripts with uncorrected *P* < .01 were included in pathway analysis. This analysis, using less stringent statistical parameters, revealed 182 and 84 significantly up- or downregulated differentially expressed genes (DEGs), respectively (Fig. 1A and 1B and Data File 1 (23)). Of these 266 DEGs, the top 10 up- and downregulated DEGs (ranked by magnitude of change in response to bed rest) and the top 25 DEGs (ranked by significance from pre to post bed rest) are presented in Fig. 1C and 1D, respectively. Transcripts involved in adipose tissue remodeling and expansion, including *MMP1* (Fig. 1C), *KIT*, and *APLN* (Fig. 1D) were significantly upregulated.

To investigate the potential pathways impacted by bed rest, KEGG and GO analyses were performed on DEGs. No pathways were enriched that associated with significantly downregulated genes. The only upregulated KEGG pathway was related to the cell cycle, while GO molecular functions also identified an enrichment for several functions related to cell division, proliferation, and mitosis, which was further supported by the enrichment of transcripts in related cellular compartments (GO-CC) (Fig. 1E). Collectively, pathway analyses suggest bed rest may promote increased cellular turnover, tissue remodeling, and proliferation/ division.

### Adipose Tissue–Insulin Signaling Pathway Responses to Bed Rest

Fasted plasma insulin significantly increased with bed rest by 1.5 ± 0.7-fold (Fig. 2A), whereas fasted blood glucose concentration was significantly reduced to 0.92 ± 0.07-fold of pre bed rest levels (Fig. 2B). Despite the change in fasting insulin, bed rest did not significantly impact the expression of any metabolic transcripts measured by qPCR in adipose tissue (all *P* > .05; Fig. 2C), which is also supported by RNA-seq results (Data File 1 (23)). There was no effect of bed rest on proteins related to insulin signaling and glucose uptake measured in whole adipose tissue or in isolated primary adipocytes (all *P* > .05; Fig. 2D and 2E).

### Inflammatory and Metabolic Proteins in Plasma and Adipose Tissue Ex Vivo Culture Explants

Plasma adipsin and adiponectin decreased 10% and 23% with bed rest, respectively, whereas leptin and resistin increased 63% and 6%, respectively (all *P* < .05). There were also substantial increases for a range of chemokines (MIP-1α, MIP-1β, MIP-3α, and IP-10; +20-66%) and osteopontin (+13%) (all *P* < .05). Significant decreases in the concentration of IL-10, IL-17C, GM-CSF, and VEGF-D were also evident following bed rest (−9 to −23%; all *P* < .05; Fig. 3A). In contrast to bed rest–induced changes detectable in plasma, none of the proteins measured in media from adipose tissue explants cultured ex vivo were significantly affected by bed rest (Fig. 3B).

The absolute change in plasma leptin was significantly positively associated with the absolute change in plasma insulin (*r* = 0.493, *P* = .027; Figure 7 (23)).

### Immunological Responses to Bed Rest in Peripheral Blood and Adipose Tissue

Adipose tissue flow cytometry measurements were available from *n* = 7 to 15 participants depending on cell type, due to either lack of tissue or contamination with peripheral blood from the biopsy procedure. There was no effect of bed rest on CD4+ and CD8+ T-cell numbers and naïve/ memory subpopulations in adipose tissue (Fig. 4A). There were no differences in adipose tissue myeloid and nonhematopoietic cell numbers, including endothelial cells and adipocyte progenitor cells, in response to bed rest (Fig. 4B-4I). Similarly, in peripheral blood, bed rest had no effect on the number of CD4+ and CD8+ T-cells and naïve/memory subpopulations or monocyte numbers (Fig. 4J-4M). However, HLA-DR expression (as a marker of activation) was significantly lower following bed rest among naïve (NA) CD4+ T-cells (Fig. 5A).

### No Effect of an Anti-inflammatory Nutrient Cocktail on Adipose Tissue During Bed Rest

The nutritional cocktail received by half of participants had no clear or consistent effects compared with the responses exhibited by the Control group. Of the 93 individual comparisons between groups in response to bed rest (excluding RNA sequencing analyses), there were only 3 statistically significant (and divergent) interaction effects following post hoc Bonferroni correction. These effects included plasma IL-17B and adipocyte insulin receptor protein content (which increased with bed rest in the Control group but not the Cocktail group), and plasma adipsin (which decreased to a greater extent with bed rest in the Cocktail group than in the Control group). This small number of isolated directionally divergent statistically significant interaction effects (~3% of all observations, excluding RNA sequencing) is unlikely to be physiologically meaningful. The nutritional cocktail also had little impact on the absolute number of transcripts differentially expressed in response to bed rest, and no clear grouping aligned with Control/Cocktail allocation following hierarchical clustering analysis (Figure 8 (23)). All data separated by group are presented in Tables 4–6 and Figures 8 and 9 (23).

## Discussion

This is the first study to examine how long-term physical inactivity (60 days bed rest) impacts human adipose tissue. We show that, when energy intake is matched to energy expenditure and fat mass is stable, adipose tissue does not exhibit profound metabolic or inflammatory changes at the protein, cellular, or transcriptional level with chronic physical inactivity. However, we also report a considerable change in circulating adipokines following bed rest which may represent both adaptive and compensatory mechanisms in response to the change in physical activity and/or energy intake.

The intervention used in the present study resulted in typical changes caused by bed rest. Lean mass was reduced by ~3 kg, which is similar to that reported previously for bed rest studies of this duration (40-42). Furthermore, fasted plasma insulin was markedly elevated following bed rest, indicating reduced whole-body insulin sensitivity with chronic physical inactivity, as consistently reported (10, 11). Thus, our novel characterization of adipose tissue responses to bed rest come from a study that is representative of the typical changes induced by this form of chronic physical inactivity. Importantly, fat mass was relatively stable across bed rest in the present study (within 1 kg) through the reduction of energy intake to match expenditure (22).

Bed rest only impacted 51 genes within the entire adipose tissue transcriptome after applying FDR corrections to DESeq2 analyses, while removing FDR corrections and applying a significance threshold of *P* < .01 on a gene by gene basis only resulted in 266 DEGs (<0.5% of the entire transcriptome). Likewise, targeted qPCR assessing a range of metabolic transcripts revealed similar results. Thus, it appears that chronic physical inactivity from bed rest does not profoundly or systematically impact adipose tissue at the transcriptional level in young healthy men when fat mass is maintained. Pathway analysis on non-FDR-corrected differentially expressed genes identified a significant enrichment of processes related to cell proliferation, division, and growth. For example, apelin, which is involved in adipocyte differentiation and stimulated by exposure to insulin (43), was significantly upregulated following bed rest, suggesting a possible stimulation of the adipogenic program in response to bed rest in adipose tissue.

Using multiparameter flow cytometry we also characterized the cellular landscape of adipose tissue in response to bed rest, including T-cells, macrophages, NK cells, and nonhematopoietic cells. The infiltration of inflammatory leukocytes into adipose tissue is a hallmark of tissue dysfunction in the context of obesity and ageing (1, 30), possibly secondary to metabolic dysregulation (44). There were no changes in the numbers of immune and nonhematopoietic cells (ie, pan-progenitor, adipocyte progenitor, and endothelial cells) in adipose tissue in response to bed rest. Given that RNA sequencing approaches used here also showed no enrichment of transcripts associated with immunological/inflammatory parameters, these results indicate that chronic physical inactivity seems not to impact adipose tissue immune cell populations in young men when fat mass is maintained.

We identified a significant increase in plasma chemokines following bed rest, including MIP-1α, MIP-1β, MIP-3α, and IP-10. A previous 2-month bed rest study reported similar chemokine responses with increased circulating IP-10 and eotaxin (CCL11) (45). In the present study, of the 32 cytokines measured, plasma IL-10 and IL-17C were reduced. The source and cause of changes to circulating chemokines and cytokines remains unclear. We did not see changes in monocyte and CD4+ and CD8+ T-cell numbers (and their respective subsets) in peripheral blood, nor changes to the secretion of these chemokines and cytokines from adipose tissue explants. The reduction in IL-10 could be caused by lower IL-6 production due to reduced skeletal muscle contraction, since IL-6 is known to stimulate IL-10 (46, 47). However, previous bed rest studies have reported no change in circulating IL-10 (45, 48), and future work should corroborate the present findings and identify which cells or tissues are the source of these altered circulating proteins following bed rest.

We observed a considerable and robust alteration to the circulating level of several adipokines, including a 63% increase in plasma leptin concentration following bed rest. Leptin normally circulates in proportion to fat mass (49), and so this large increase in plasma leptin without a major change in fat mass is particularly striking. Leptin has myriad roles at the tissue and whole-body level, influencing lipid and carbohydrate metabolism, body mass homeostasis, and appetite regulation (50-55). The increase in circulating leptin probably reflects heightened leptin secretion from an existing intracellular pool within adipocytes, secondary to the increase in plasma insulin (56-58). Leptin secretion is known to be influenced by insulin, independent of fat mass (59), and the positive relationship between the change in plasma insulin and leptin supports the notion that insulin is a key driver behind the observed leptin response. The implications of this large increase in leptin remain to be established, including whether it is associated with altered leptin sensitivity, but such a large increase in leptin could contribute to prior reports of appetite dysregulation with low levels of physical activity (60, 61).

Although we report significantly elevated circulating leptin, we did not detect changes in short-term ex vivo secretion of leptin from adipose tissue explants following bed rest. Leptin is secreted from adipose tissue in vast quantities, at high rates of release (62), and it has a short half-life (<1 hour) in the circulation (63). Consequently, such substantial increases in plasma leptin following bed rest, without alterations to ex vivo secretion, is ostensibly counterintuitive. However, the in vivo local adipose tissue environment will have changed considerably with bed rest, whereas the ex vivo adipose tissue explant environment (ie, media composition) remained constant pre and post bed rest. Therefore, the lack of change in ex vivo leptin secretion reported in the present study, despite substantially elevated circulating levels, may reflect the removal of signals (such as insulin) that were driving the increase in leptin secretion in vivo. Thus, we propose that intrinsic basal adipose tissue leptin secretion is not fundamentally changed with chronic physical inactivity, but that leptin secretion is increased secondary to upstream mediators or other in vivo changes (eg, insulin-induced adipose tissue glucose uptake).

In contrast to leptin, circulating plasma adiponectin significantly declined with bed rest. Adiponectin promotes insulin sensitivity at the whole-body and skeletal muscle level (52) and acts locally on adipocytes to increase glucose uptake, independent of the actions of insulin (64, 65). Further, adiponectin concentration increases in parallel to adipocyte insulin sensitivity (66). Given the role of adiponectin as an insulin-sensitizing adipokine (52), the reduction in circulating adiponectin might represent a homeostatic readjustment due to elevated insulin. Similarly to leptin, the change in adiponectin does not appear to be intrinsic or irreversible given that secretion from adipose tissue cultured ex vivo was unaffected by bed rest.

The present study is the first investigation of adipose tissue responses to long-term physical inactivity (bed rest). We only included males and the distribution and function of adipose tissue differs substantially between the sexes (67, 68), so the impact of long-term physical inactivity on adipose tissue in females remains unknown. We also acknowledge that, while this was a long-term bed rest study of 60 days, chronic physical inactivity in real-world settings could span years or decades. Thus, it is possible that the healthy, young volunteers recruited in the present study were able to maintain their adipose tissue phenotype for 2 months, which may not be the case with years of physical inactivity. However, this hypothesis is difficult to test experimentally given that 60 days of bed rest is already considered a long and demanding manipulation of human physical activity.

In conclusion, this study demonstrates that, when energy balance is maintained and fat mass is kept stable, adipose tissue does not exhibit profound metabolic or inflammatory changes at the protein, cellular, or transcriptional level after 2 months of bed rest. However, there were considerable changes to the concentration of circulating adipokines with bed rest, such as leptin and adiponectin, that were not apparent under standardized ex vivo conditions. Thus, we conclude that altered circulating adipokine concentrations reflect changes in adipose tissue adipokine secretion secondary to other changes such as increased circulating insulin concentrations. These adipose tissue responses to physical inactivity may have important implications for appetite control and the regulation of insulin sensitivity in real-world settings where energy intake and balance is not so tightly controlled.

## Data Availability

Supplementary data cited in this work can be accessed at Figshare (23) (https://doi.org/10.6084/m9.figshare.15138231). Sequencing data sets associated with this work are available from GenBank (GSE1822303). All remaining datasets generated during the current study are available in the University of Bath Data Repository (69) (https://doi.org/10.15125/BATH-01052).

## Abbreviations

BMC: bone mineral content
BMR: basal metabolic rate
BP: biological process
CC: cellular compartment
CCL: C-C motif ligand
CD: cluster of differentiation
CM: central memory
CNES: centre national d’etudes spatiales
CXCL: C-X-C motif ligand
DAVID: database for annotation visualization, and integrated discovery
DEG: differentially expressed gene
DEXA: dualenergy X-ray absorptiometry
EASE: expression analysis systematic explorer
EM: effector memory
EMRA: effector memory re-expressing CD45RA
ESA: european space agency
FDR: false discovery rate
FGF: fibroblast growth factor
GM-CSF: granulocyte macrophage colony stimulating factor
GO: gene ontology
HLA-DR: human leukocyte antigen-DR isotype
ICAM-1: intercellular adhesion molecule-1
IFN-γ: interferon gamma
IL: interleukin
IP-10: interferon gamma-induced protein 10
KEGG: kyoto encyclopedia of genes and genomes
MCP: monocyte chemoattractant protein
MEDES: médecine et de physiologie spatiales
MF: molecular function
MFI: median fluorescence intensity
MIP: macrophage inflammatory protein
MSCA-1: mesenchymal stem cell antigen-1
NA: naive
PBMC: peripheral blood mononuclear cell
qPCR: quantitative polymerase chain reaction
RANTES: regulated on activation, normal T cell expressed and secreted
RIN: ribosomal integrity number
SAA: serum amyloid-A
SVF: stromal vascular fraction
TNF: tumor necrosis factor
VCAM-1: vascular cell adhesion molecule-1
VEGF: vascular endothelial growth factor

## Additional Information

**Disclosures:** The authors declare no competing interests.
